# Efficacy of transoral fundoplication for treatment of chronic gastroesophageal reflux disease incompletely controlled with high-dose proton-pump inhibitors therapy: a randomized, multicenter, open label, crossover study

**DOI:** 10.1186/1471-230X-14-174

**Published:** 2014-10-06

**Authors:** Karim Sami Trad, Gilbert Simoni, William Edris Barnes, Ahmad Bassel Shughoury, Mamoon Raza, Jeffrey Alan Heise, Daniel Gilles Turgeon, Mark Alan Fox, Peter George Mavrelis

**Affiliations:** Department of Surgery, The George Washington University School of Medicine and Health Sciences, Washington DC, USA; Reston Surgical Associates, Reston, VA USA; Advanced Gastroenterology, Inc, Thousand Oaks, CA USA; Department of Surgery, Livingston Hospital and Healthcare Services, Inc. CAH, Salem, KY USA; Department of Gastroenterology, Saint Mary Medical Center, Hobart, IN USA; Internal Medicine Associates, Merrillville, IN USA; Indiana Medical Research, Elkhart, IN USA; Department of Gastroenterology, Unity Surgical Hospital, Mishawaka, IN USA; Heartburn Center/Rehabilitation Department, Hancock Regional Hospital, Greenfield, IN USA; Crossville Medical Group, Crossville, TN USA; Department of Surgery, Cumberland Medical Center, Crossville, TN USA

**Keywords:** TIF, EsophyX, Atypical GERD symptoms, Regurgitation

## Abstract

**Background:**

The aim of this randomized, crossover study was to determine if transoral fundoplication (TF) could further improve clinical outcomes in partial responders to high-dose (HD) proton-pump inhibitor (PPI) therapy and to evaluate durability of TF.

**Methods:**

In seven United States centers, patients with hiatal hernia ≤2 cm and abnormal esophageal acid exposure (EAE) were randomized to TF (n = 40) or HD PPIs (n = 23) group. At 6-month follow-up, PPI patients underwent crossover. We assessed clinical outcomes 6-month post TF in crossover patients (COP), as compared to 6-month of HD PPI therapy, and 12-month outcomes in patients initially randomized to TF. The primary outcome was symptom control evaluated by Reflux Disease Questionnaire and Reflux Symptom Index. Secondary outcomes included healing of esophagitis, normalization of EAE and PPI use after TF. We analyzed 21 COP and 39 TF patients. McNemar’s test or Fisher exact test was used to compare proportions.

**Results:**

Of 63 randomized patients, 3 were lost to follow-up, leaving 39 TF and 21 COP for analyses. In the COP, TF further improved control of regurgitation and of atypical symptoms achieved after six months of HD PPIs. Of 20 patients with GERD symptoms after six months of high-dose PPI therapy, 65% (13/20) reported global elimination of troublesome regurgitation and atypical symptoms post TF off PPIs; 67% (6/9) reported no troublesome regurgitation. Esophagitis further healed in 75% (6/8) of patients. Seventy-one percent of COP patients were off PPIs six months following TF. Normalization of EAE decreased from 52% after HD PPIs (on PPIs) to 33% after TF (off PPIs), p =0.388. In the original TF group, 12-month post TF, 77% of patients achieved complete symptom control, 82% ceased PPI therapy, 100% healed esophagitis and 45% normalized EAE.

**Conclusions:**

The results of this study indicate that in patients with incomplete symptom control on high-dose PPI therapy TF may provide further elimination of symptoms and esophagitis healing. In the original TF group, the clinical outcomes of TF remained stable between 6- and 12-month follow-up.

**Trail registration:**

Clinicaltrials.gov: NCT01647958.

**Electronic supplementary material:**

The online version of this article (doi:10.1186/1471-230X-14-174) contains supplementary material, which is available to authorized users.

## Background

Gastroesophageal reflux disease (GERD) is a chronic condition afflicting millions of Americans on a daily basis [1]. Despite major medical therapeutic advances in the past three decades, treatment alternatives remain imperfect for a portion of patients suffering from persistent troublesome symptoms, as defined by the Montreal consensus statement, while on daily high-dose proton-pump inhibitors (PPIs) therapy [2, 3]. Laparoscopic fundoplication, while currently considered the gold standard alternative to medical therapy, has seen decreased popularity mostly due to recurrent symptoms requiring medical therapy and the associated troublesome side effects such as dysphagia and gas bloat [4, 5]. PPI therapy, while the most common and effective treatment for patients with moderate to severe heartburn, provides unsatisfactory or incomplete responses to 20-40% of patients, mostly those suffering from regurgitation or extra-esophageal manifestations of GERD [6, 7].

The EsophyX device (EndoGastric Solutions, Inc., Redmond, WA) is one of several endoluminal platforms developed to offer a less invasive procedural alternative to treat GERD [8]. The transoral fundoplication (TF) technique has combined symptomatic success with an acceptable safety profile and few post fundoplication side effects [9]. While TF appears to be an attractive alternative to current medical and surgical therapies in select patients, there are no randomized crossover studies evaluating TF in patients who respond incompletely to high-dose PPIs.

The TEMPO (TF EsophyX vs. Medical PPI Open Label Trial) randomized crossover trial was designed with a dual aim; (1) to compare the efficacy of TF to high-dose PPI in patients with troublesome symptoms of GERD and (2) to investigate the effectiveness of the TF procedure used as a cross over strategy in patients who remained incompletely treated on high-dose PPIs. Preliminary six month results comparing two parallel treatment arms of this trial were reported elsewhere [10]. For the present study, we hypothesized that TF would enhance the therapeutic effects achieved by high-dose PPI therapy in the same group of patients (crossover group), and that the clinical outcomes of TF would remain stable over time in patients initially randomized to the TF group.

## Methods

### Study design and patients

This prospective, comparative, randomized study with a crossover group enrolled 63 patients between June and August 2012 at seven institutions in the United States. Included were patients with daily troublesome regurgitation and/or atypical GERD symptoms (Montreal criteria) on PPIs, abnormal 48-hour ambulatory pH test defined as% time pH <4 greater than 5.3% of the total recording period [11] and a history of daily PPI use for at least six months. We excluded patients with a body mass index (BMI) greater than 35 kg/m^2^, Los Angeles grade C or D esophagitis [12], hiatal hernia >2 cm in either dimension, Hill grade valve III or IV [13, 14] and Barrett’s esophagus >2 cm. The study was approved by the institutional review board of each participating institution and appropriately registered (clinicaltrials.gov: NCT01647958) [10]. Western Institutional Review Board approved the study at the following centers: The George Washington University School of Medicine and Health Sciences, Washington, District of Columbia; Reston Surgical Associates, Reston, Virginia; Advanced Gastroenterology, Inc., Thousand Oaks, California; Livingston Hospital and Healthcare Services, Inc. CAH, Salem, Kentucky; Saint Mary Medical Center, Hobart, Indiana; Internal Medicine Associates, Merrillville, Indiana; Indiana Medical Research, Elkhart, Indiana; Unity Surgical Hospital, Mishawaka, Indiana; Heartburn Center/Rehabilitation Department, Hancock Regional Hospital, Greenfield, Indiana; Crossville Medical Group, Crossville, Tennessee and Cumberland Medical Center, Crossville, Tennessee. Additionally, due to the local regulatory requirements, the Reston Hospital Center Institutional Review Board approved the study at The George Washington University School of Medicine and Health Sciences, Washington, District of Columbia and Reston Surgical Associates, Reston, Virginia. The participating centers were led by gastroenterologists (3) and surgeons (4). All patients provided written informed consent before randomization.

In our community settings, the public at large and some institutional review boards were reluctant to include a sham procedure arm in our study design. Considering the previously published results of the safety and effectiveness of TF and taking into account the concerns about subjecting study patients to unnecessary risks associated with a sham procedure, we employed a non-blinded study design for this post market, phase IV randomized trial.

### Pretreatment evaluation and randomization

Baseline data, while on PPIs, were collected during the initial visit using three standardized tools: (i) Reflux Disease Questionnaire (RDQ), (ii) Reflux Symptom Index (RSI) and (iii) Gastroesophageal Health-related Quality of Life (GERD-HRQL). Patients who reported daily troublesome regurgitation and/or atypical symptoms on daily PPIs underwent esophagogastroduodenoscopy (EGD) and 48-hr pH monitoring after a ‘wash out’ period of at least seven days. Symptom assessment was repeated off PPIs using the same GERD questionnaires. Barium swallow and esophageal motility were used selectively when clinically indicated.

Randomization was done by computer-generated block sequence with stratification according to participating centers. The size of randomization blocks was nine. Patients were randomly assigned to be treated either with TF or high dose PPI targeting a 2(TF):1(PPI) ratio via sealed opaque envelopes. Patients who met the eligibility criteria were informed of the treatment allocation in the office by opening the envelopes provided by an independent statistician in their presence.

### Treatments

In the TF group, investigators used an EsophyX-2 device to create a partial esophago-gastric fundoplication following the previously described TIF 2.0 protocol [15]. Briefly, the device is introduced over a flexible endoscope and inserted into the stomach under constant endoscopic visualization. The device and endoscope are retroflexed. The helical retractor is engaged into the tissue just below the Z line. The fundus of the stomach is then folded up and wrapped around the distal esophagus utilizing the tissue mold, the chassis and the helix as an anchor. After locking all the tissue manipulating elements, the invaginator is activated to allow the separation of the gastroesophageal junction from the diaphragm. The polypropylene “H” fasteners are then delivered through the tissue. The maneuver is repeated at three additional positions to create a full thickness, partial gastro-esophageal fundoplication. An additional movie file shows the TF procedure in more details [see Additional file 1]. An intraoperative endoscopy under general anesthesia is performed immediately before introducing the device to confirm Hill grade and size of hiatal hernia.

After TF, patients were kept overnight for observation. Patients were instructed to stop PPI therapy two weeks after TF, to follow the standard post-fundoplication diet and to refrain from vigorous physical activities for the first 6 weeks.

In the PPI group, patients were instructed to take high-dose PPI therapy splitting the dose into twice daily before breakfast and dinner. For the purpose of this study, we defined high-dose PPI therapy as the maximal allowed dose set by the manufacturers, as spelled out in the labeled indications.

### Follow-up and crossover

At 6-month follow-up, all patients completed symptom assessment using the same GERD specific questionnaires. Patients in the TF group underwent EGD and 48-hr pH metry off PPIs for at least 7 days. Patients in the PPI group underwent objective testing on high-dose PPI therapy. After completing 6-month visit, patients from the original PPI group underwent TF (crossover group). Patients from the original TF group underwent the same assessments at 12-month follow-up visit. We are reporting on the potential additional therapeutic effects of TF in a crossover population who had achieved a partial response to dose escalation of PPI therapy. Additionally, we are reporting the clinical outcomes at 12-month follow-up in patients initially randomized to the TF group.

### Outcomes

The primary endpoint was elimination of daily troublesome regurgitation or atypical symptoms. Clinical success was defined by the elimination of troublesome regurgitation per Montreal consensus definition [3], as evaluated by the RDQ questionnaire. The elimination of daily troublesome atypical symptoms was assessed by the RSI questionnaire (each individual atypical score ≤2) score. Secondary endpoints included PPI use, healing of reflux esophagitis and normalization of esophageal acid exposure (EAE).

Heartburn, dysphagia and bloating were assessed using GERD-HRQL; excess flatulence was assessed with a standalone question. We also performed correlation analyses to measure the degree of association between subjective and objective (pH) outcomes. Finally, we performed univariate and multivariate logistic regression analyses to detect preoperative factors associated with normalization of EAE.

### Statistical methods

Statistical analysis was performed using JMP 10.0 (SAS, Cary, North Carolina) statistical program. With a sample size of 28 (TF) and 14 (PPIs) patients, a two group Fisher exact test would have a power of 80% to demonstrate a significant difference between groups. Under the assumption that more than 70% of the patients allocated to the TF group would have their daily troublesome symptoms eliminated compared to ≤20% in the PPI group, and a two-sided α error of 0.05, the predetermined sample size would provide an 80% power to detect a significant difference between the two groups. Results are presented as means (standard deviation) or percent (counts). Comparison of means was done with analysis of variance with post-hoc Tukey’s Honesty Significant Difference test. As appropriate, McNemar’s test or Fisher exact test was used to compare proportions. Relationship between post-treatments pH outcomes and patient reported subjective outcomes were estimated using Spearman’s rank correlation (rho). Univariate and stepwise backward multivariate logistic regression analyses were performed to examine associations between preoperative pH parameters and normalization of EAE. A p value of less than 0.05 was considered significant. All authors had access to the study data and reviewed and approved the manuscript.

## Results

**Figure 1:**
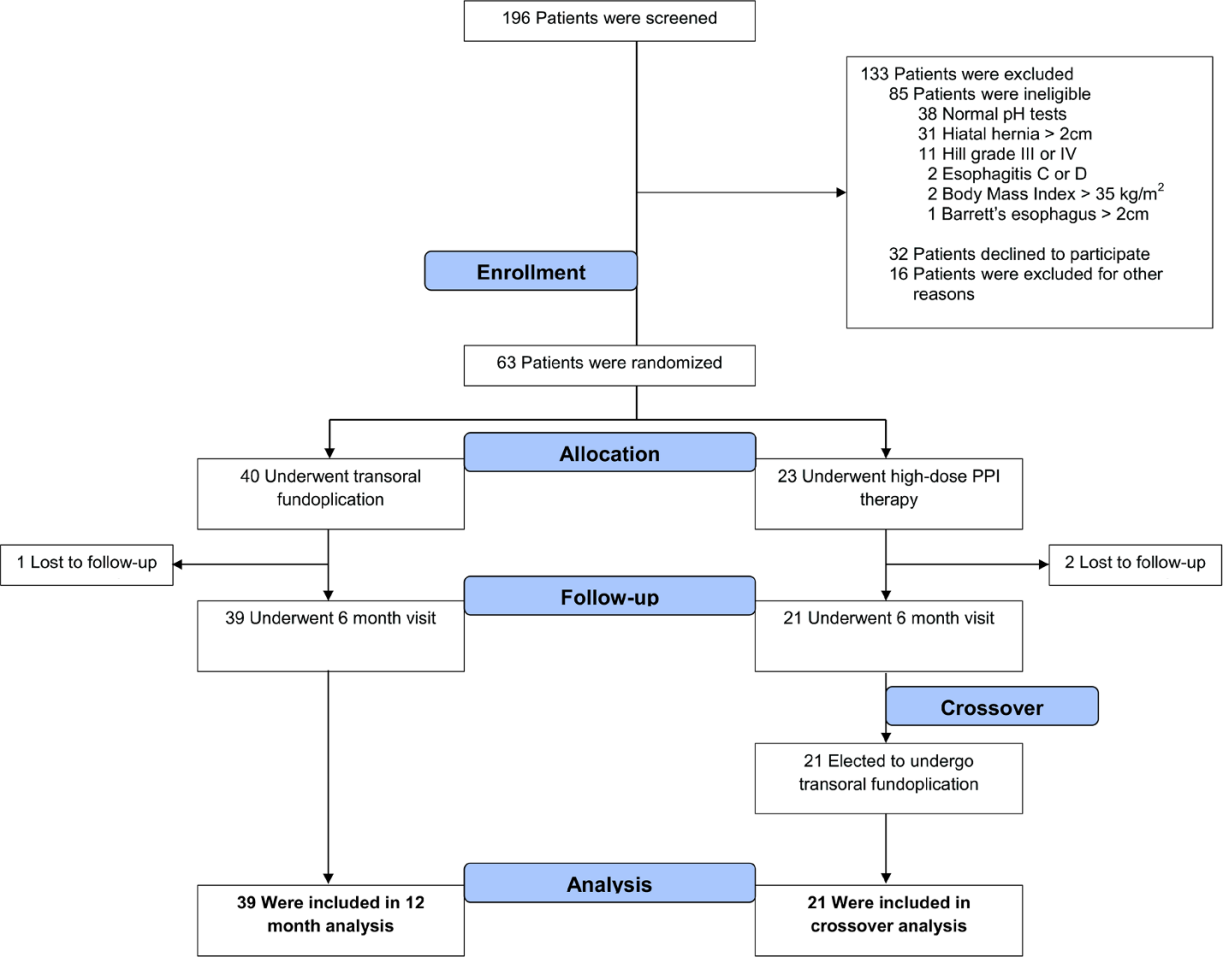
**CONSORT diagram of the study.**

Among 196 chronic GERD patients referred to our institutions for an antireflux procedure, 32% (63/196) were randomized (Figure 1). Baseline characteristics of enrolled patients were balanced between the TF and PPI group, including proportion of patients who were on high-dose PPI therapy at screening; 31% of TF patients versus 24% of PPI patients (Table 1). There were no reports of serious adverse events associated with the TF procedure in either group. The procedure data are presented in Table 2.

**Table 1:** **Demographics and clinical characteristics of the study patients at screening**

Characteristics	TF group (n = 39)	PPI group (n = 21)	p values
Male, n (%)	19 (49)	8 (38)	0.587
Age, years	54.5 (10.4)	50.4 (9.8)	0.151
Body mass index, kg/m^2^	28.3 (3.9)	29.1 (2.8)	0.422
GERD symptom duration, years	12.5 (11.3)	9.4 (5.7)	0.224
PPI therapy duration, years	9.3 (7.2)	8.1 (5.1)	0.686
Quality of life scores off PPIs			
Reflux Disease Questionnaire (RDQ) score	3.7 (1.1)	4.1 (0.7)	0.122
Regurgitation (RDQ) score off PPIs	3.5 (1.2)	4.1 (0.9)	0.067
GERD-HRQL score	32.5 (9.6)	33.7 (8.5)	0.625
Heartburn score (GERD-HRQL)	22.2 (6.3)	23.2 (4.4)	0.519
Reflux Symptom Index (RSI) score	25.2 (9.3)	28.6 (7.4)	0.150
Total % time pH <4	10.2 (3.5)	10.6 (3.5)	0.665
Patients on single dose of PPI at entry, n (%)	27 (69)	16 (76)	0.765

GERD, gastroesophageal reflux disease; GERD-HRQL, gastroesophageal reflux disease health related quality of life; PPI, proton pump inhibitor; TF, transoral fundoplication.

**Table 2:** **Procedure data**

Parameters	TF group (n = 39)	Crossover group (n = 21)	p values
Fasteners used, mean (range)	21 (16–30)	20 (10–30)	0.786
Valve length (cm), mean (range)	2.8 (2.5-4)	2.9 (2.5-4)	0.894
Valve circumference (degrees), mean (range)	290 (240–340)	300 (270–300)	0.647
Hiatal hernia reduced, n (%)	36 (100)	16 (100)	> 0.999
Hill grade I achieved (from Hill grade II), n (%)	31 (100)	18 (100)	> 0.999
Length of stay >24 hours, n (%)	2 (5)	1 (5)	> 0.999

Immediate post-operative endoscopy was used to assess hiatal hernia and Hill grade.

TF, transoral fundoplication.

### Crossover patients (6-month follow-up)

#### Primary endpoint

Six months after TF, 65% [13/20, 95% confidence interval (CI), 43 to 82] of patients reported global elimination of troublesome regurgitation and any atypical symptoms off PPI therapy. One patient who reported global elimination of GERD symptoms after high-dose PPI therapy was excluded from this analysis. Of 9 patients who reported troublesome regurgitation after being on high-dose PPI therapy for six months, 67% (6/9, 95% CI, 35 to 88) reported no troublesome regurgitation six months following TF while off PPIs. TF, as compared to high-dose PPIs, further improved quality of life scores and control of various atypical symptoms such as hoarseness, throat cleaning, postnasal drip and troublesome or annoying cough (Table 3).

**Table 3:** **Mean quality of life scores at screening (on PPIs), six months after high-dose proton-pump inhibitor (PPIs) and six months after** ***crossover*** **to transoral fundoplication (TF) in patients initially randomized to the PPI group**

Parameters	Screening (on PPIs)	6-month on high-dose PPIs	6-month after TF (off PPIs)	p value (6-month PPIs vs. screening)	p value (6-month TF vs. screening	p value (6-month PPIs vs. 6-month TF)
Reflux disease questionnaire (RDQ)	3.04 (0.99)	2.14 (1.20)	1.33 (1.76)	0.089	< 0.001	0.136
Heartburn (RDQ)	3.14 (1.31)	1.89 (1.37)	1.57 (1.96)	0.033	0.006	0.787
Dyspepsia (RDQ)	2.95 (1.21)	2.05 (1.31)	1.44 (1.92)	0.137	0.006	0.400
Regurgitation (RDQ)	3.02 (1.09)	2.46 (1.28)	0.98 (1.72)	0.398	< 0.001	0.003
Gastroesophageal reflux disease health-related quality of life (GERD-HRQL)	26.43 (7.22)	18.86 (9.12)	10.05 (13.54)	0.053	< 0.001	0.020
Heartburn (GERD-HRQL)	16.90 (5.75)	11.67 (6.94)	7.48 (9.81)	0.078	< 0.001	0.189
Reflux Symptom Index	22.62 (8.33)	19.62 (7.29)	8.76 (11.02)	0.151	< 0.001	< 0.001
Hoarseness	2.19 (1.75)	2.29 (1.42)	0.95 (1.50)	0.979	0.034	0.020
Throat clearing	2.90 (1.23)	2.90 (0.92)	1.23 (1.51)	> 0.999	< 0.001	< 0.001
Excess throat mucus or postnasal drip	3.48 (1.14)	2.95 (0.84)	1.43 (1.56)	0.368	< 0.001	< 0.001
Difficulty swallowing foods, liquids or pills	2.14 (1.28)	1.19 (1.18)	0.48 (0.96)	0.029	< 0.001	0.129
Coughing after eating or after lying down	2.47 (1.57)	2.29 (1.35)	0.86 (1.53)	0.909	0.002	0.008
Breathing difficulties or chocking episodes	1.57 (1.50)	1.19 (1.08)	0.38 (0.86)	0.550	0.005	0.075
Troublesome or annoying cough	2.14 (1.49)	2.33 (1.53)	0.90 (1.45)	0.910	0.025	0.008
Sensation or something sticking or a lump in the throat (globus)	2.48 (1.44)	1.71 (1.10)	1.10 (1.76)	0.216	0.009	0.359
Heartburn, chest pain, indigestion or stomach acid coming up	3.24 (1.30)	2.76 (1.30)	1.43 (1.86)	0.566	< 0.001	0.016
Excess flatulence	2.62 (1.75)	2.24 (1.45)	0.70 (1.38)	0.702	< 0.001	0.006

Values represent means (standard deviations). PPI, proton pump inhibitor; TF, transoral fundoplication.

#### Secondary endpoints

**Figure 2:**
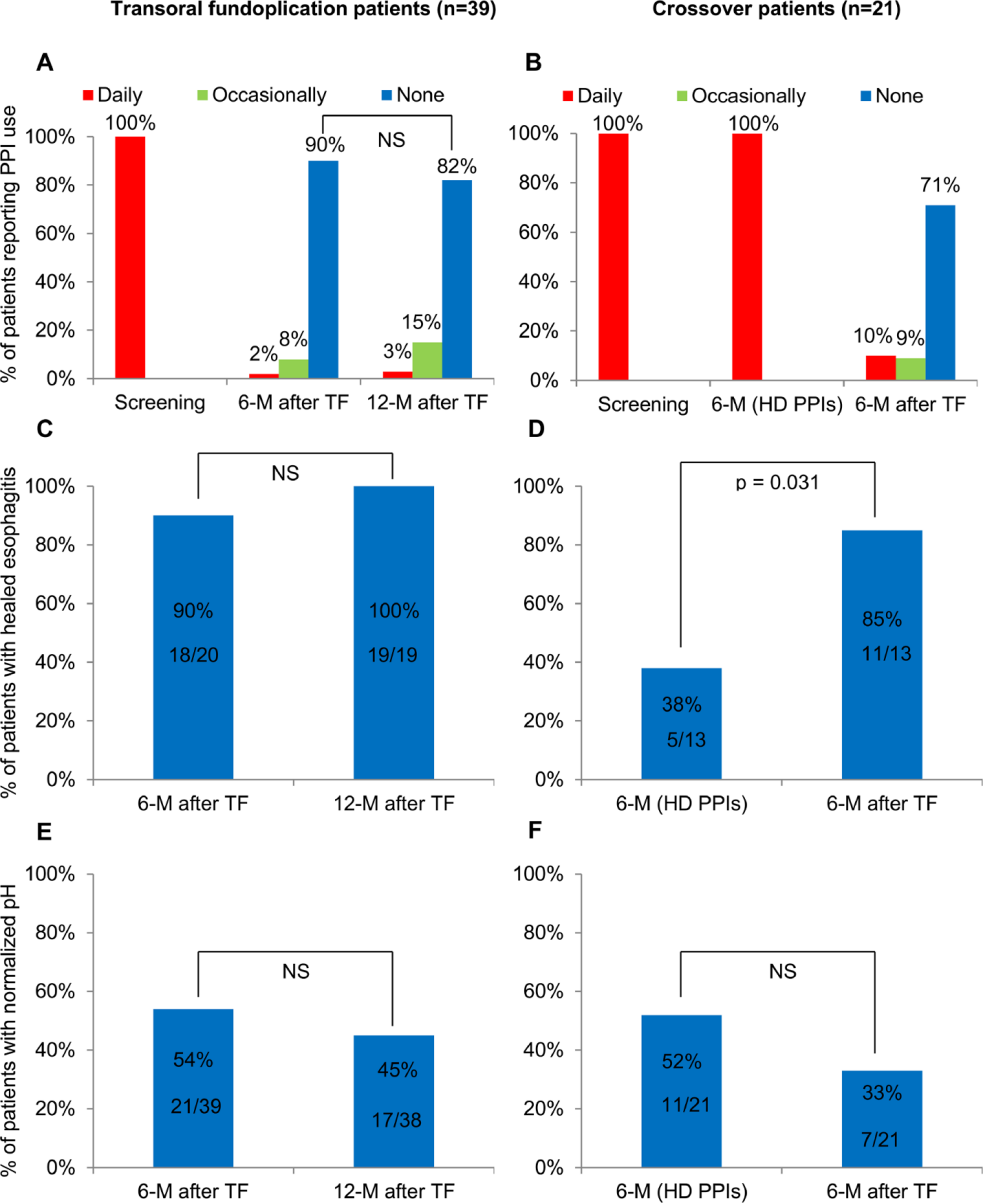
**Proton pump inhibitors use (A and B), healing of reflux esophagitis (C and D) and normalization of esophageal acid exposure (E and F) in both treatment groups at different study intervals.**

PPI use, healing of reflux esophagitis and pH normalization in the crossover group are shown in Figure 2. Of six patients who were back on PPI therapy after TF, 67% (4/6, 95% CI, 30 to 90) reduced their dose ≥50% while 33% (2/6, 95% CI, 10 to 70) remained on high-dose PPI therapy. Esophageal pH parameters at entry, six months after high-dose PPI therapy and six months after TF in crossover patients are presented in Table 4.

**Table 4:** **48-hour pH parameters through the phases of the study in patients initially randomized to the high-dose proton-pump inhibitors (PPIs) group (A) and to the transoral fundoplication (TF) group (B)**

A Parameters	Screening (off PPIs)	6-month on high-dose PPIs	6-month after TF (off PPIs)	p value (6-month PPIs vs. screening)	p value (6-month after TF vs. screening)	p value (PPIs vs.TF)
Number of refluxes	190.43 (89.36)	91.33 (68.01)	120.52 (64.25)	< 0.001	0.010	0.420
Number of long refluxes (>5 minutes)	12.52 (7.22)	4.62 (5.07)	10.00 (6.02)	< 0.001	0.387	0.017
Duration of longest reflux, minutes	26.48 (14.29)	23.76 (21.77)	24.86 (14.71)	0.867	0.950	0.977
Fraction time pH <4, %	10.50 (3.51)	5.01 (4.55)	7.87 (4.20)	< 0.001	0.070	0.104
DeMeester score	35.79 (13.05)	19.29 (16.07)	28.60 (14.71)	0.002	0.258	0.108
**B Parameters**	**Screening (off PPIs)**	**6-month (off PPIs)**	**12-month (off PPIs)**	**p value (6-month vs. screening)**	**p value (12-month vs. screening)**	**p value (6-month vs. 12-month)**
Number of refluxes	160.72 (76.71)	100.80 (83.58)	115.26 (61.28)	0.002	0.023	0.672
Number of long refluxes (>5 minutes)	12.62 (5.48)	8.54 (7.68)	10.24 (7.89)	0.030	0.297	0.536
Duration of longest reflux, minutes	31.36 (14.15)	23.41 (20.75)	23.82 (15.04)	0.010	0.128	0.994
Fraction time pH <4, %	10.19 (3.55)	6.77 (5.41)	7.45 (4.86)	0.005	0.031	0.800
DeMeester score	35.28 (11.80)	23.64 (18.54)	25.32 (15.50)	0.004	0.016	0.883

Values represent means (standard deviation). PPI, proton pump inhibitor; TF, transoral fundoplication.

#### Ancillary analyses

De novo dysphagia, bloating or excess flatulence was not reported in crossover patients six months post TF. Statistically significant correlations between regurgitation, atypical symptoms and pH parameters were not found six months after high-dose PPI therapy and six months after TF (Table 5). A moderate correlation between GERD-HRQL score and number of reflux episodes on high-dose PPIs was found six months after high-dose PPI therapy.

**Table 5:** **Correlation between objective (pH) and patient reported subjective outcomes in study patients PPI group on PPIs (6 months following high-dose PPI therapy)**

Parameters	Regurgitation	RSI	GERD-HRQL	DMS	% total time	RE
Regurgitation	1.00					
RSI	0.70 (<0.001)	1.00				
GERD-HRQL	0.59 (0.005)	0.71 (<0.001)	1.00			
DMS	−0.20 (0.399)	0.05 (0.838)	0.38 (0.107)	1.00		
% total time	−0.12 (0.619)	0.12 (0.598)	0.41 (0.063)	0.94 (<0.001)	1.00	
RE	0.10 (0.667)	0.31 (0.167)	0.46 (0.035)	0.77 (<0.001)	0.84 (<0.001)	1.00
**Crossover patients off PPIs (6 months following transoral fundoplication)**						
**Parameters**	**Regurgitation**	**RSI**	**GERD-HRQL**	**DMS**	**% total time**	**RE**
Regurgitation	1.00					
RSI	0.71 (<0.001)	1.00				
GERD-HRQL	0.81 (<0.001)	0.62 (0.003)	1.00			
DMS	0.18 (0.428)	0.14 (0.556)	0.04 (0.866)	1.00		
% total time	0.11 (0.637)	0.09 (0.705)	0.08 (0.746)	0.89 (<0.001)	1.00	
RE	0.17 (0.461)	0.14 (0.546)	0.14 (0.543)	0.65 (0.001)	0.80 (<0.001)	1.00
**TF group off PPIs (12 months following transoral fundoplication)**						
**Parameters**	**Regurgitation**	**RSI**	**GERD-HRQL**	**DMS**	**% total time**	**RE**
Regurgitation	1.00					
RSI	0.66 (<0.001)	1.00				
GERD-HRQL	0.42 (0.010)	0.73 (<0.001)	1.00			
DMS	0.02 (0.885)	0.10 (0.550)	0.35 (0.036)	1.00		
% total time	0.06 (0.724)	0.10 (0.552)	0.39 (0.018)	0.98 (<0.001)	1.00	
RE	0.21 (0.216)	0.13 (0.424)	0.41 (0.013)	0.83 (<0.001)	0.84 (<0.001)	1.00

Values are Spearman’s Rho (p values).

DMS, DeMeester score; GERD-HRQL, gastroesophageal reflux disease health-related quality of life; PPI, proton pump inhibitor; RE, reflux episodes; RSI, Reflux Symptom Index.

Regurgitation was assessed with Reflux Disease questionnaire. RSI was used to assess atypical symptoms. GERD-HRQL was used to assess typical symptoms.

### TF group (12-month follow-up)

#### Primary endpoint

At 12-month follow-up off PPIs, global elimination of any atypical symptoms and regurgitation was achieved in 77% (30/39, 95% CI, 62 to 87) of patients; elimination of troublesome regurgitation was achieved in 93% (28/30, 95% CI, 79 to 98). Elimination of all atypical symptoms was experienced in 82% (32/39, 95% CI, 67 to 91) of patients. There was no significant change in the various QOL scores at different study intervals (Table 6).

**Table 6:** **Mean quality of life scores before transoral fundoplication (TF) and at 6- and 12- month follow-up in patients initially randomized to the transoral fundoplication (TF) group**

Parameters	Baseline (Before TF on PPIs)	6-month (off PPIs)	12-month (off PPIs)	p value (6-month vs. baseline)	p value (12-month vs. baseline)	p value (6-month vs. 12-month)
Reflux disease questionnaire (RDQ)	2.91 (1.32)	0.35 (0.53)	0.50 (0.73)	< 0.001	< 0.001	0.772
Heartburn (RDQ)	2.99 (2.55)	0.45 (0.86)	0.63 (1.01)	< 0.001	< 0.001	0.776
Dyspepsia (RDQ)	2.81 (1.57)	0.42 (0.83)	0.53 (0.88)	< 0.001	< 0.001	0.917
Regurgitation (RDQ)	2.94 (1.45)	0.19 (0.40)	0.33 (0.69)	< 0.001	< 0.001	0.791
Gastroesophageal reflux disease health-related quality of life (GERD-HRQL)	26.25 (10.51)	5.23 (7.14)	5.41 (6.80)	< 0.001	< 0.001	0.995
Heartburn (GERD-HRQL)	17.69 (7.51)	3.74 (5.51)	3.76 (4.50)	< 0.001	< 0.001	> 0.999
Reflux Symptom Index	22.00 (9.63)	4.64 (5.53)	4.79 (6.67)	< 0.001	< 0.001	0.995
Hoarseness	1.76 (1.56)	0.18 (0.60)	0.33 (0.90)	< 0.001	< 0.001	0.810
Throat clearing	2.90 (1.37)	0.87 (1.28)	0.72 (1.10)	< 0.001	< 0.001	0.851
Excess throat mucus or postnasal drip	2.77 (1.51)	0.87 (1.20)	0.79 (1.06)	< 0.001	< 0.001	0.961
Difficulty swallowing foods, liquids or pills	1.92 (1.44)	0.33 (0.77)	0.46 (0.88)	< 0.001	< 0.001	0.858
Coughing after eating or after lying down	2.46 (1.54)	0.44 (1.02)	0.44 (0.88)	< 0.001	< 0.001	> 0.999
Breathing difficulties or choking episodes	1.85 (1.60)	0.13 (0.41)	0.33 (0.96)	< 0.001	< 0.001	0.690
Troublesome or annoying cough	2.21 (1.49)	0.49 (1.19)	0.54 (1.12)	< 0.001	< 0.001	0.983
Sensation or something sticking or a lump in the throat (globus)	2.74 (1.41)	0.41 (0.94)	0.44 (0.75)	< 0.001	< 0.001	0.994
Heartburn, chest pain, indigestion or stomach acid coming up	3.38 (1.39)	0.92 (1.22)	0.87 (1.26)	< 0.001	< 0.001	0.983
Excess flatulence	2.36 (1.65)	0.72 (1.19)	0.68 (1.1)	< 0.001	< 0.001	0.990

Values represent means (standard deviations).

#### Secondary endpoints

PPIs use, healing of reflux esophagitis and normalization of esophageal acid exposure through the duration of the study is shown in Figure 2. All esophageal pH parameters at 12-month follow-up were significantly reduced compared to baseline (Table 4).

#### Ancillary analyses

There were no reports of de novo dysphagia or bloating at 12-month follow-up; one patient reported de novo excess flatulence (from score 0 at screening on PPIs to score 3 off PPIs at 12-month follow-up).

In the TF group, correlation analyses between patient reported subjective outcomes and pH outcomes off PPIs, showed moderate statistically significant correlation between GERD-HRQL scores (typical symptoms) and objective outcomes at 12-month follow-up (Table 5). As with the crossover group, statistically significant correlations between pH parameters, regurgitation and atypical symptoms were not found.

### Association analysis

For the purpose of the association analysis, we increased our sample size by combining TF patients from the original treatment group with crossover patients. Preoperative total number of long refluxes <12 (p <0.001), DeMeester score <35 (p =0.011),% total time ≤10 (p =0.013) and absence of esophagitis (p =0.035) were positively associated with normalization of EAE on univariate analyses. However, on a multivariate level, only preoperative total number of long refluxes <12 stayed significantly associated with normalization of EAE (odds ratio 9.6, 95% CI 2.8 to 39.0, p <0.001). When this factor was favorable, normalization of EAE in the TF group increased from 45% to 65% (15/23, 95% CI, 45 to 81) at 12-month follow-up. When this factor was unfavorable (≥12), pH normalization was achieved in only 13% (2/15, 95% CI, 4 to 38) of TF patients. In crossover patients, when a number of long refluxes were favorable, pH normalization was reached in 56% (5/9, 95% CI, 27 to 81) of patients, six months following TF. When a number of long refluxes was ≥12, pH normalization was achieved in 17% (2/12, 95% CI, 5 to 45) of crossover patients.

## Discussion

This report is the first to specifically investigate the effectiveness of TF in a controlled *crossover* design in patients with partial control of regurgitation and extraesophageal symptoms on high-dose PPI therapy. In the crossover group of patients, six months of high-dose PPI therapy provided measurable symptomatic improvement (Table 3), while TF further improved control of a range of GERD symptoms, particularly regurgitation and atypical symptoms. This is an important finding of this study. Patients with typical GERD symptoms who demonstrate good response to PPI therapy are often considered to be the best candidate for anti-reflux procedures [6]. Findings from this study suggest that well selected patients (small hiatal hernia, Hill grade I or II and esophagitis less than grade C) with an incomplete response to dose escalation of PPIs could also benefit from TF. It appears that in this study TF was better than high-dose PPIs in the global elimination of regurgitation and all atypical symptoms (65% six months after TF vs. 5% [10] six months of high-dose PPIs). For this analysis, we elected to utilize a crossover design rather than analyzing two parallel groups to eliminate any potential confounding factors that may influence clinical outcomes.

The overall response of regurgitation to PPI therapy has been estimated to be about 17% greater than placebo and >20% less than that observed for heartburn [7]. In the crossover group, 67% (6/9) of patients who reported persistent troublesome regurgitation despite six months of high-dose PPI therapy experienced complete elimination of regurgitation six months following TF. This demonstrates a notable gain in a challenging patient population and may well have important therapeutic implications. We believe that the ability of TF to eliminate regurgitation is primarily due to the correction of anatomic defects at the gastro-esophageal junction [10].

Additionally, this report demonstrates that TF is capable of achieving a sustained control of regurgitation and a range of atypical symptoms for at least 12 months post-procedure (Table 6). In the TF group, the proportion of patients reporting elimination of troublesome regurgitation was stable between 6-month (97%) [10] and 12-month (93%) follow-up. We also noted that the proportion of patients reporting elimination of all atypical symptoms increased from 62% at 6-month [10] to 82% at 12-month, a finding in line with previous studies suggesting that atypical symptoms tend to resolve at a slower pace than typical symptoms after anti-reflux surgery [16]. We suspect that the proportion of crossover patients free of atypical symptoms 6-month post TF will increase at 12-month follow-up as has been the case for patients in the original TF group.

Complete discontinuation of acid-suppressive medications has been a common end point in many studies evaluating surgical or endoscopic anti-reflux therapy. Even occasional intake of PPIs to suppress GERD symptoms following anti-reflux procedures has been often viewed as a treatment failure [17]. Of 7 patients (18%) who continued taking any dose of PPIs 12 months following TF, elimination of troublesome regurgitation was reported in 80% (4/5) of patients; elimination of troublesome atypical symptoms was achieved in 57% (4/7) of patients. We believe that controlling troublesome symptoms and improving QOL with the use of PPIs after an endoscopic anti-reflux procedure in patients who suffered from *uncontrolled* troublesome symptoms on high-dose PPIs before intervention should not necessarily be considered a treatment failure. This is the case in a sizable proportion of patients in our study. TF may therefore be viewed as a useful therapeutic adjunct to PPIs. Pre-procedure patient counseling is mandatory in setting appropriate expectations regarding the eventual need for PPI use after TF.

A significant correlation between postoperative pH parameters, atypical symptoms and regurgitation were not found in either treatment arm. A poor correlation between postoperative GERD symptoms and physiological parameters has been reported previously after traditional anti-reflux surgery [18, 19]. Data on the correlation between symptomatic relief and pH parameters after high-dose PPI therapy are sparse. In one prospective study, 50% of asymptomatic GERD patients on PPI therapy still presented with abnormal pH study with 75% of studied patients taking double-dose PPIs [20]. The most recent study from Stanford University found that 54% of asymptomatic GERD patients on PPI therapy still had abnormal esophageal pH profiles [21]. This may suggest that PPI therapy may be less efficacious in controlling abnormal esophageal acid exposure in certain patients than previously thought. We speculate that the higher prevalence of non-acid reflux or weekly acid reflux in our population may explain the low rate of healing of reflux esophagitis in the PPI group [10]. The authors believe that the poor correlation between post-treatment symptom control and pH data in both symptomatic and asymptomatic patients warrants an effort to further define the role and significance of pH testing in this setting.

High rates of symptomatic relief and healing of reflux esophagitis after TF in this study were not matched by equivalent rates of distal esophageal pH normalization. Symptomatic relief may be more important than pH normalization for some patients suffering from mild to moderate symptoms and who are reluctant to undergo more invasive surgical treatment options. We noted a statistically insignificant decline in the proportion of patients who normalized distal EAE post TF (from 54% at 6- to 45% at 12-month). On the other hand, symptom control improved and healing of reflux esophagitis remained stable between two follow-up intervals. In attempting to reconcile these apparently contradictory findings, we speculate that the majority of patients in this study may have suffered from non-acid reflux or experienced excessive proximal extent of reflux episodes [10]. Additionally, there may be a potential for investigator bias toward the assessment of endoscopic findings. The authors strongly suggest that future studies regarding treatments for GERD utilize pH impedance testing.

A recent report from the Maastricht University [22] suggests that TF reduces the number of postprandial transient lower esophageal sphincter relaxations (TLESRs) and the number of TLESRs associated with reflux. TF also is shown to significantly reduce the distensibility of the gastro-esophageal junction. It appears that the effect of TF is selective for liquid-containing reflux episodes while the number of gas reflux episodes remains unaffected, suggesting preservation of the ability of venting gas following TF [22]. Although we were not able to confirm these important findings in our study since impedance testing was not used, we believe that the findings from the Maastricht University accurately present the mechanisms of the antireflux effect of TF. In our view, the consistent absence of post-fundoplication symptoms associated with TF reported in previous studies [9], and confirmed in the current study, represents one of the most attractive element of the TF procedure.

In this study, preoperative Hill grade (I or II) did not affect post-operative clinical outcomes. Patients with more severe anatomic defect (Hill grade III and IV) [23, 24] and more severe erosive disease [25] are less likely to respond favorably to TF and were excluded from our study.

The TF procedure has demonstrated an acceptable safety record. An exhaustive review of the published literature suggests a 3.2% incidence of serious adverse events associated with the TF procedure [9]. In our study, the complete absence of any serious adverse events such as esophageal tears, perforations or significant bleeding requiring transfusion is likely due to the experience of the investigators who were all past their learning curve for the TF (each performed more than 20 TF cases before the study initiation) . This also suggests that proper technique and safe handling of the device could help to further reduce incidence of complications.

There were some limitations to our study. First, this was an open-label, pre-planned crossover, non-blinded trial which may carry a certain unintended bias. Second, although symptomatic control in the TF group improved at 12-month compared to 6-month, a residual placebo effect could still have impacted the reported results. Third, the lack of pH impedance testing and systematic high-resolution manometry data prevented us from clarifying the presumed effects of TF on patients with non-acid and proximal reflux, and on number of TLESRs. Finally, for our association analysis, we combined the TF and the crossover patient to increase the sample size. We believe that these analyses should be repeated on a larger patient population.

We focused our association analysis on normalization of EAE since very few patients presented with recurring GERD symptoms after TF. Our findings suggest that pH normalization following TF could be improved by selecting appropriate patients with favorable preoperative objective characteristics. In this study, several factors were positively associated with normalization of EAE on univariate level (preoperative total number of long refluxes <12, DeMeester score <35,% total time ≤10 and absence of esophagitis; however, on a multivariate level, only preoperative total number of long refluxes <12 stayed significantly associated with normalization of EAE. Although preoperative high resolution manometry was selectively performed, we do not have any indications to believe that this finding was due to poor preoperative esophageal motility. In fact, the increased number of long reflux events is a significant driver of increased esophageal acid exposure (personal communication Dr. Kahrilas). Furthermore, long reflux events occur more common in the supine position and are mainly a function of hiatal hernia size. We suspect that relatively low number of long reflux events (<12) as a predictive factor of normalization of EAE may suggest that a large proportion of patients without hiatal hernia or very small hiatal hernia may achieve normalization of EAE. Plans are being made to perform a comprehensive analysis of preoperative factors influencing post procedure normalization of EAE on a large number of patients.

The patient population under study was characterized by a mixed symptomatology with an incomplete response to PPIs, which is representative of a clinical challenge commonly encountered in the community setting. A previous report had also noted that the patient populations in the majority of TF studies are skewed towards those with the most severe GERD symptoms [9]. Our study achieved notable results in controlling symptoms and healing of reflux esophagitis compared to high-dose PPIs. One may speculate that even better results are possible in patients with less severe, more homogeneous symptomatology. Perhaps future studies could focus on the patient population with typical symptoms who completely respond to PPI therapy and who have traditionally been considered preferred candidates for anti-reflux surgery.

## Conclusions

In conclusion, the results of this randomized study suggest that in patients with small hiatal hernias and an incomplete response to high-dose PPI therapy, TF can further improve on the therapeutic effects achieved with high-dose PPIs. It would appear that high-dose PPI therapy provides better control of the number of long refluxes while other pH parameters are similarly improved with both therapies. Additionally, TF can safely provide sustained control of regurgitation, atypical symptoms and healing of reflux esophagitis for at least 12 months, without risking the development of post-fundoplication side effects.

## Electronic supplementary material

Additional file 1: **Transoral Fundoplication Procedure Video.** (ZIP 11 MB)

## Abbreviations

BMI: Body mass index
COP: Crossover patients
EAE: Esophageal acid exposure
EGD: Esophagogastroduodenoscopy
HD: High-dose
GERD: Gastroesophageal reflux disease
GERD-HRQL: Gastroesophageal reflux disease health-related quality of life
PPI: Proton pump inhibitor
QOL: Quality of life
RDQ: Reflux disease questionnaire
RSI: Reflux Symptom Index
TEMPO: TF EsophyX vs. medical PPI open label trial
TF: Transoral fundoplication
TLESRs: Transient lower esophageal sphincter relaxations
WA: Washington.
